# Comparing General Anaesthesia With Spinal Anaesthesia for Holmium Laser Enucleation of Prostate: A Systematic Review and Meta-Analysis

**DOI:** 10.7759/cureus.117086

**Published:** 2026-09-28

**Authors:** Shahzad Ahmad, Angus Bruce, Tharani Nitkunan

**Affiliations:** 1 Urology, Epsom and St Helier University Hospitals NHS Trust, London, GBR

**Keywords:** benign prostatic hyperplasia (bph), endo urology, general anaesthesia, holmium laser enucleation of prostate (holep), spinal anaesthesia

## Abstract

Holmium laser enucleation of the prostate (HoLEP) is an established surgical treatment for benign prostatic hyperplasia (BPH). Although it is conventionally performed under general anaesthesia (GA), spinal anaesthesia (SA) may represent an important alternative for patients with significant medical comorbidity. We performed a systematic review and meta-analysis comparing peri-operative outcomes of HoLEP performed under GA versus SA.

A systematic review and meta-analysis was conducted in accordance with Preferred Reporting Items for Systematic Reviews and Meta-Analyses (PRISMA) guidelines. A comprehensive search of electronic databases was performed (MEDLINE, PubMed, EMBASE and Google Scholar) for comparative studies evaluating GA versus SA for HoLEP. A random-effects meta-analysis was performed using mean difference (MD) or odds ratio (OR) with 95% confidence intervals (CI). A search of electronic databases found four studies, enrolling a total of 2323 patients.

SA was associated with a significantly shorter operative time than GA (P=0.03), although this finding was not robust on leave-one-out sensitivity analysis. There were no significant differences in resected tissue weight (P=0.73), Hb drop (P=0.07), post-operative catheter reinsertion (P=0.34), Clavien-Dindo grade ≥3 complications (P=0.14), change in IPSS (P=0.91), change in QMax (P=0.60), or change in PVR (P=1.00). Moderate to high heterogeneity was observed for several outcomes, and the available evidence was limited to retrospective and propensity-matched studies.

Current evidence suggests that spinal anaesthesia could be a safe and effective alternative to general anaesthesia for HoLEP, with comparable intra- and post-operative outcomes. SA may expand access to HoLEP for patients at increased risk from GA. However, the available evidence remains limited, and high-quality prospective randomised studies are required to confirm these findings.

## Introduction and background

Benign prostatic hyperplasia (BPH) continues to increase in prevalence in an ageing population and represents a major cause of bothersome lower urinary tract symptoms (LUTS) [1-3]. A range of surgical interventions have been developed to address symptomatic BPH, among which holmium laser enucleation of the prostate (HoLEP) has emerged as a well-established, safe, and effective treatment option [4,5]. Importantly, HoLEP demonstrates durable efficacy across a broad spectrum of prostate sizes [6,7].

In addition to BPH, elderly patients franequently develop age-related comorbidities, including coronary artery disease and chronic obstructive pulmonary disease, which may increase the physiological risks associated with general anaesthesia (GA) [8,9]. Although HoLEP is conventionally performed under GA, this approach may be unsuitable for certain patients with significant medical comorbidities. In such cases, spinal anaesthesia (SA) poses a potential alternative, avoiding some peri-operative anaesthetic risks associated with GA [10-12].

While comprehensive reviews and meta-analyses exist comparing GA and SA for other treatment modalities, such as transurethral resection of the prostate (TURP), we are aware of no comprehensive reviews comparing the intra- and post-operative outcomes of HoLEP when using GA versus SA.

In view of this, we aimed to perform a systematic review and meta-analysis to compare intra- and post-operative HoLEP outcomes, comparing GA to SA.

## Review

Materials and methods

This systematic review and meta-analysis has been performed in accordance with the Preferred Reporting Items for Systematic Reviews and Meta-Analyses (PRISMA) statement standards [13]. Prospective registration in the International Prospective Register of Systematic Reviews was obtained (CRD420261392335).

Eligibility Criteria

Our inclusion criteria consisted of all comparative studies comparing the use of GA and SA for HoLEP procedures. Studies including other bladder outlet procedures were excluded. In cases where studies included HoLEP and other bladder outlet procedures, only the HoLEP subgroup was included for analysis. No exclusion criteria were made based on patient age or pre-operative prostate size. Single-arm studies, letters to the editor, or case reports were excluded.

Outcome Measures

The measured intra-operative outcomes were considered as the primary outcome measures. These included operative time, prostatic tissue resected weight, and intra-operative drop in haemoglobin (Hb).

The post-operative outcomes were considered our secondary outcome measures. These included the requirement for post-operative catheter reinsertion, Clavien-Dindo 3+ complications [14], change in International Prostate Symptom Score (IPSS) [15], change in maximum flow rate (QMax), and change in post-void residual volume (PVR). 

Search Strategy

Two independent authors performed a comprehensive search strategy of electronic databases (MEDLINE, PubMed, EMBASE, and Google Scholar). The last search was completed on May 11, 2026. The literature search strategy is outlined in the Appendices. No language limitations were applied.

Each independent author screened the title and abstract of articles to assess for suitability and relevance to our study. A third opinion was sought in the event of discrepancy in opinion. Additionally, references of included articles were screened to potentially identify further articles for inclusion. The literature search flow chart is displayed in Figure 1.

**Figure 1:**
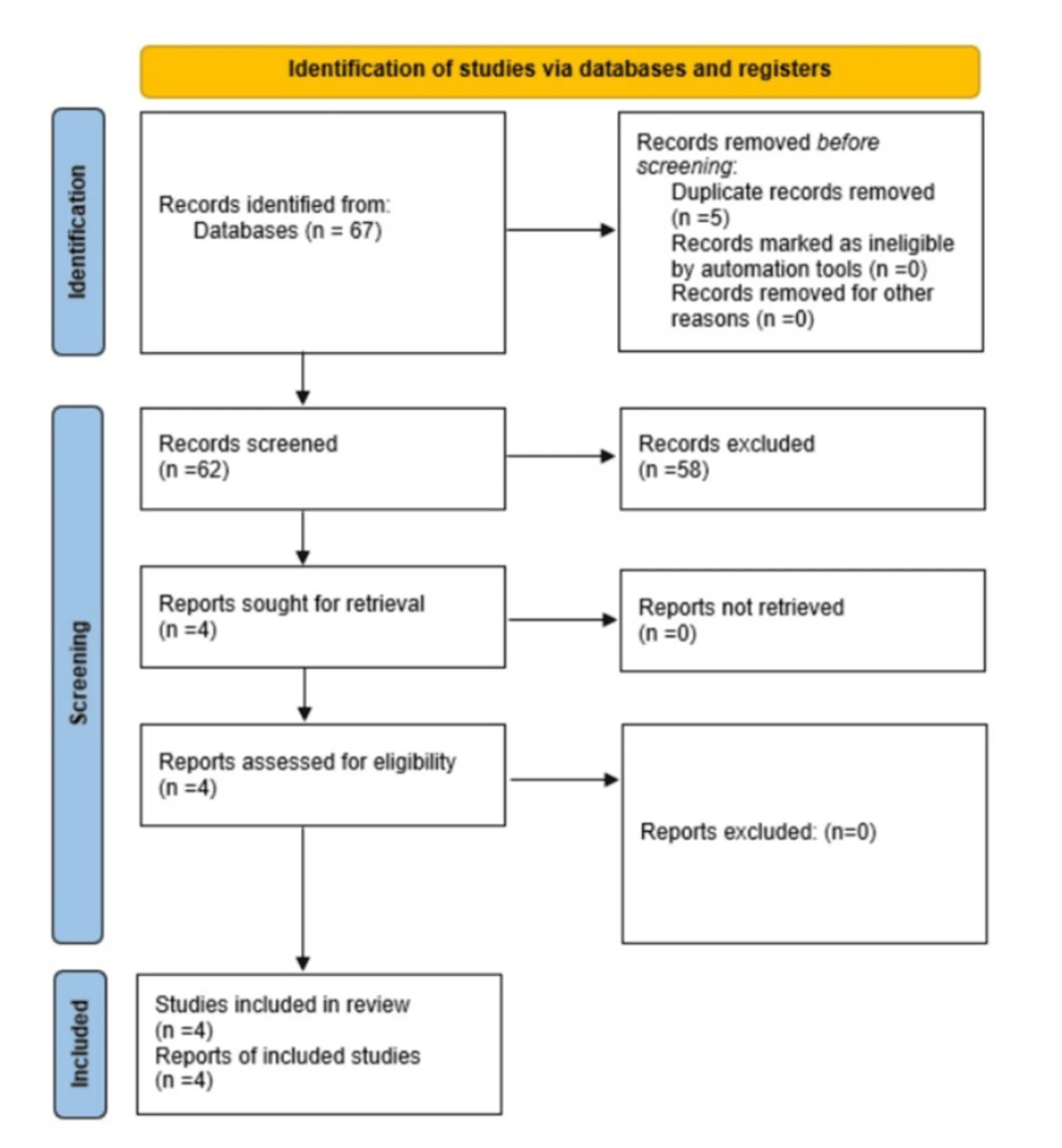
PRISMA flow diagram of the study PRISMA: Preferred Reporting Items for Systematic Reviews and Meta-Analyses

Data Extraction

An electronic data extraction spreadsheet was produced prior to data collection, in accordance with the Cochrane data collection form for intervention reviews [16]. Data were extracted regarding study-specific data, baseline demographic details (age, BMI, prostate volume, catheter dependence, PSA, anticoagulant use) and outcome data.

Summary Measures and Synthesis

For dichotomous outcome measures (need for post-operative catheter reinsertion, Clavien-Dindo 3+ complications), an odds ratio (OR) was calculated as the estimate of effect size. The OR is the odds of an adverse outcome in the GA group compared to that in the SA group. An OR of less than one would favour GA. For continuous outcome measures (operative time, resected weight, Hb drop, change in IPSS/QMax/PVR), the mean difference (MD) was calculated. When mean values were not available, data on median and interquartile range (IQR) were extracted and subsequently converted to mean and standard deviation (SD) using the established technique as described by Hozo et al. [17].

Review Manager 5.4 software (The Cochrane Collaboration, London, England, UK) was utilised for analyses, using random-effects modelling. The results of data synthesis for the evaluated outcomes were presented in a forest plot demonstrating 95% confidence intervals (CIs). Statistical heterogeneity was quantified using I2: 0-50% interpreted as low heterogeneity, 50-75% as moderate heterogeneity, 75-100% as high heterogeneity.

Further sensitivity analysis was performed to investigate potential causes for heterogeneity. Analysis was performed using a fixed-effects model, as well as leave-one-out sensitivity analysis.

Results

Search Results

Following a search of the aforementioned databases, 67 articles were found. Following review, a total of 4 comparative studies [18-21] were included, comprising a total of 2323 patients.

Table 1 demonstrates the characteristics of included studies, while Table 2 demonstrates the baseline demographics of the included populations.

**Table 1:** Characteristics of included studies GA: general anaesthesia; SA: spinal anaesthesia

Author	Journal	Country	Study design	Sample Size	GA	SA
Haehn et al., 2025 [18]	Urology	USA	Prospective propensity-matched study	622	311	311
Silvani et al., 2024 [19]	World Journal of Urology	Italy	Retrospective study	142	37	111
Westhofen et al., 2022 [20]	Urology	Germany	Retrospective study	400	200	200
Yu et al., 2023 [21]	Urologia Internationalis	Germany	Retrospective study	1159	785	374

**Table 2:** Baseline demographics of included studies GA: general anaesthesia; SA: spinal anaesthesia; BMI: body mass index; PSA: prostate specific antigen; AC: anticoagulant; AP: antiplatelet; NR: not recorded; NS: not significant (p>0.05)

Author	Sample size	GA	SA	Age (years)	BMI (kg/m²)	Prostate volume (cc)	Catheter dependence (%)	PSA (ng/mL)	AC/AP use (%)
Haehn et al., 2025 [18]	622	311	311	71±7.3 vs. 71±7.6 (p=0.3)	29±4.6 vs. 28±4.3 (p=0.01)	94±36.8 vs. 98±36.8 (p=0.9)	17.5 vs. 19 (p=0.7)	NR	NR
Silvani et al., 2024 [19]	142	37	111	71 vs. 71 (p=0.8)	26.5 vs. 26.6 (p=0.9)	80 vs. 79 (p=0.8)	32.4 vs. 32.4 (p=0.6)	3.9 vs. 4.0 (p=0.9)	39.6 vs. 40.5 (p=0.7)
Westhofen et al., 2022 [20]	400	200	200	74 vs. 74 (p=NS)	25.9 vs. 25.6 (p=NS)	75 vs. 75 (p=NS)	NR	4.3 vs. 5.2 (p=NS)	NR
Yu et al., 2023 [21]	1159	785	374	71 vs. 71 (p=0.07)	80 vs. 80 (p=0.4)	80 vs. 80 (p=0.4)	28 vs. 28 (p=0.8)	5.6 vs. 5.3 (p=0.8)	10 vs. 16 (p=0.01)

Methodological Quality and Risk of Bias

Two independent authors performed a methodological quality assessment, with a third available in the event of discrepancy. The results of the assessment are displayed in Figure 2.

**Figure 2:**
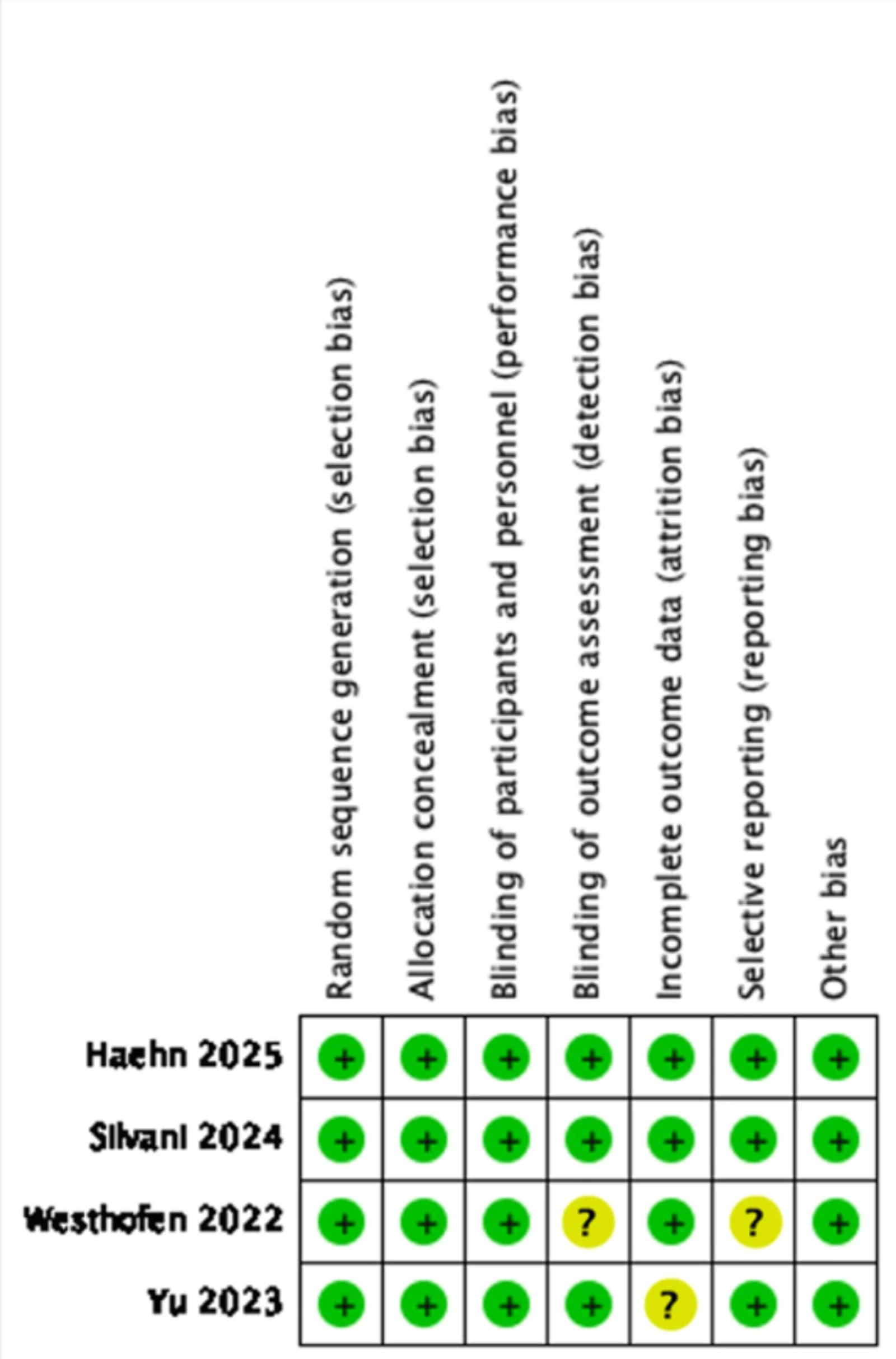
Methodological quality assessment [18-21]

Outcome Synthesis (Primary Outcomes)

Operative time: Operative time was reported in 4 studies [18-21], enrolling 2252 patients (Figure 3). SA was associated with a statistically significantly shorter operative time (MD 5.26, 95% CI 0.59 to 9.93, P=0.03). Moderate heterogeneity was detected amongst the included studies (I2=64%, P=0.04).

**Figure 3:**
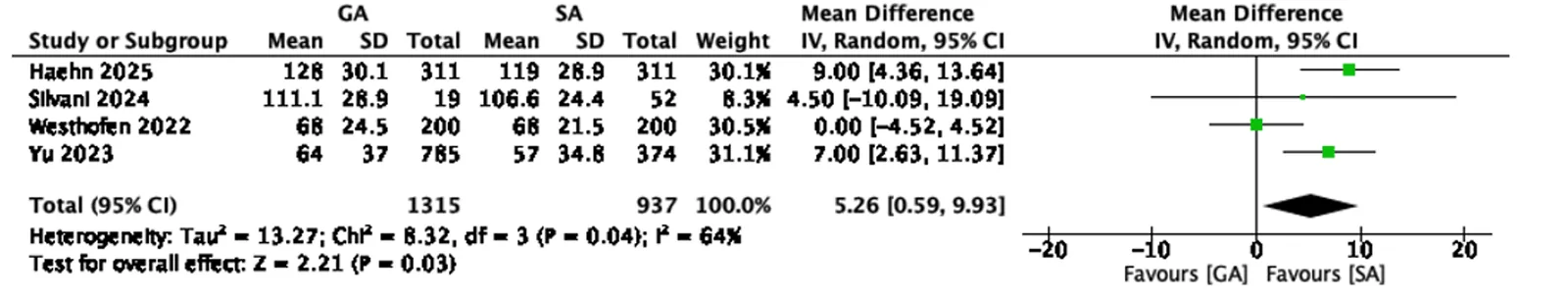
Forest plot of operative time GA: general anaesthesia; SA: spinal anaesthesia; SD: standard deviation; CI: confidence interval [18-21]

Resected weight: Prostatic resected weight was reported in 3 studies [19-21], enrolling 1630 patients (Figure 4). There was no statistically significant difference in the resected weight between the GA and SA (MD -0.57, 95% CI - 3.81 to 2.67, P=0.73). Low heterogeneity was detected amongst the included studies (I2=0%, P=0.73).

**Figure 4:**
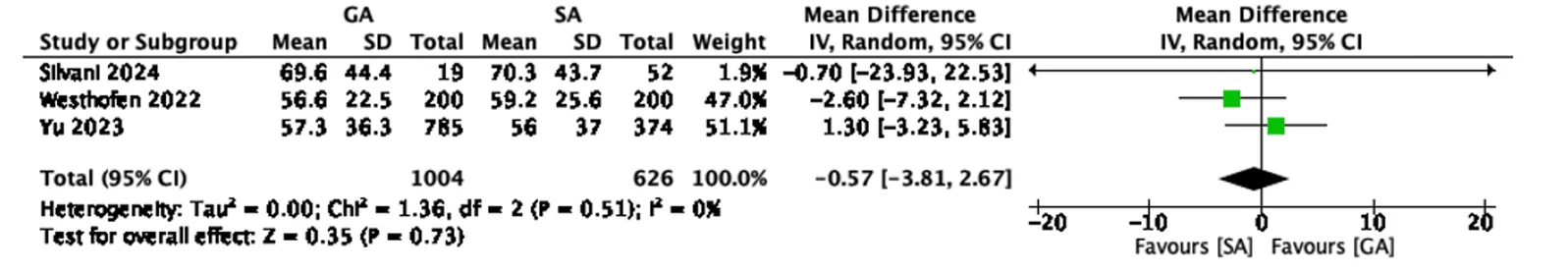
Forest plot of resected weight GA: general anaesthesia; SA: spinal anaesthesia; SD: standard deviation; CI: confidence interval [19-21]

Haemoglobin drop: An intra-operative drop in haemoglobin levels was reported in 3 studies [19-21], enrolling 1630 patients (Figure 5). There was no statistically significant difference in the haemoglobin drop between GA and SA (MD 0.15, 95% CI - 0.01 to 0.31, P=0.07). Moderate heterogeneity was detected amongst the included studies (I2=56%, P=0.10).

**Figure 5:**
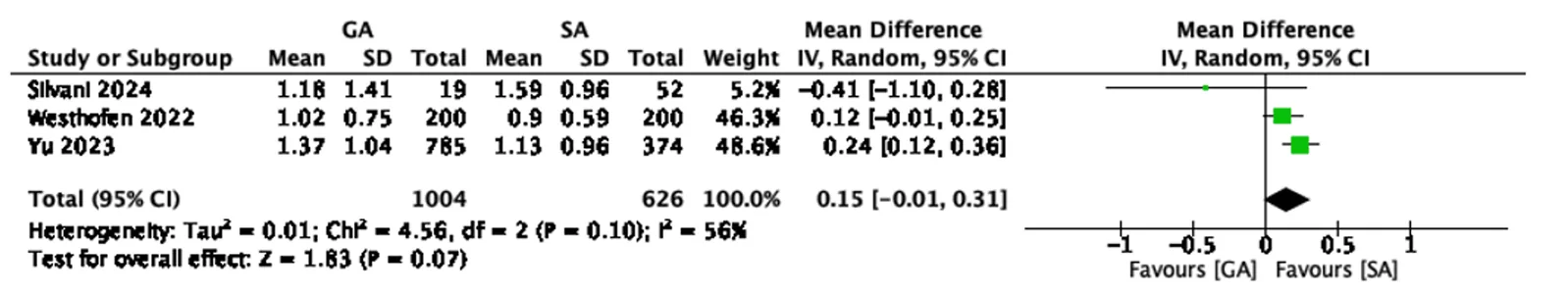
Forest plot of haemoglobin drop GA: general anaesthesia; SA: spinal anaesthesia; SD: standard deviation; CI: confidence interval [19-21]

Outcome Synthesis (Secondary Outcomes)

Heterogeneity was observed between the studies regarding timing of follow-up and, as a result, recorded post-operative IPSS, QMax and PVR. Westhofen et al. [20] reported on IPSS results 4 weeks post-operatively, Silvani et al. [19] 3 months, and Yu et al. at a median of 33 months [21].

Need for post-operative catheter reinsertion: The requirement for a post-operative catheter reinsertion was reported in 3 studies [18,19,21], enrolling 1929 patients (Figure 6). There was no statistically significant difference in the catheter reinsertion rates between GA and SA cohorts (OR 0.82, 95% CI 0.54 to 1.24, P=0.34). Low heterogeneity was detected amongst the included studies (I2=0%, P=0.79).

**Figure 6:**
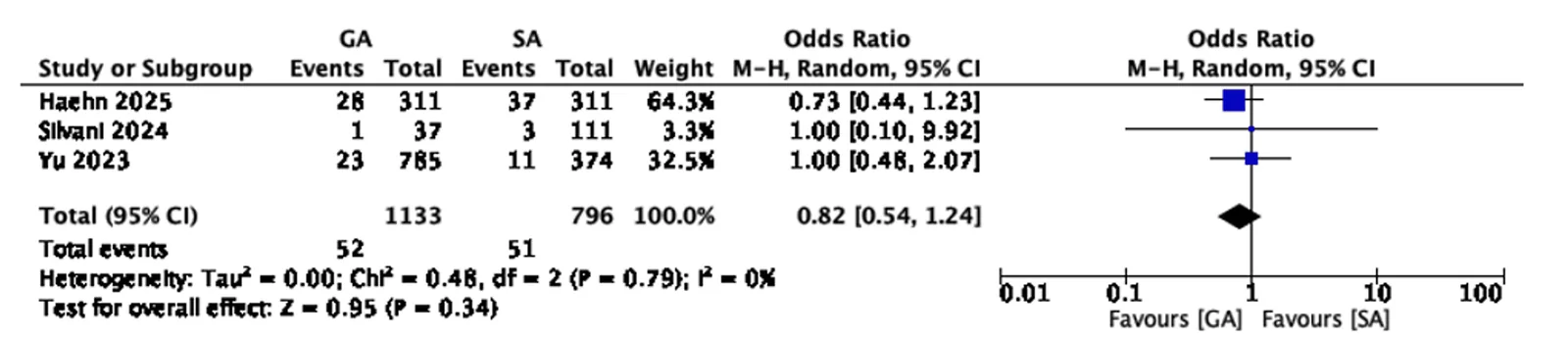
Forest plot of need for post-operative catheter insertion GA: general anaesthesia; SA: spinal anaesthesia; SD: standard deviation; CI: confidence interval [18,19,21]

Complications (Clavien-Dindo 3+): The complication rate (Clavien-Dindo 3+) was reported in 3 studies [18,20,21], enrolling 2181 patients (Figure 7). There was no statistically significant difference in complication rates between the GA and SA cohorts (OR 1.54, 95% CI 0.87 to 2.71, P=0.14). Moderate heterogeneity was detected amongst the included studies (I2=37%, P=0.21).

**Figure 7:**
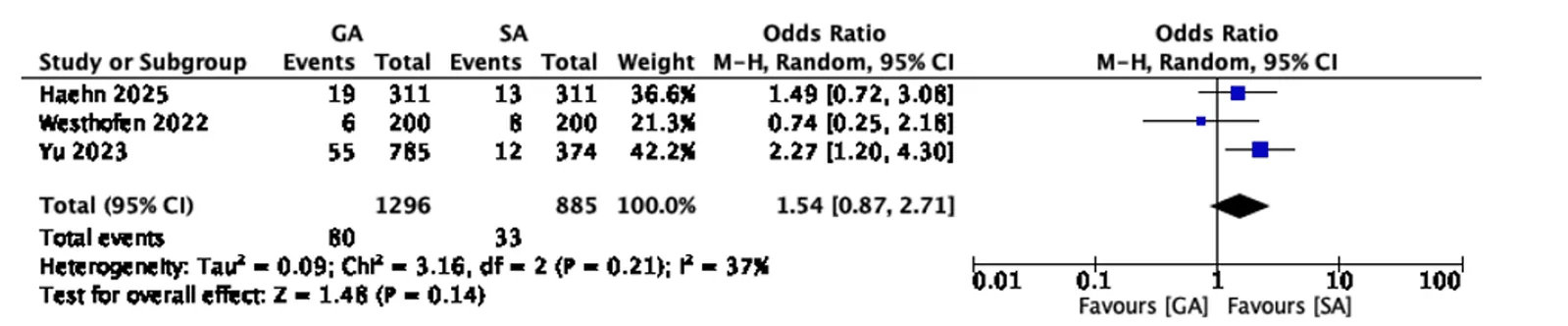
Forest plot of complications (Clavien-Dindo 3+) GA: general anaesthesia; SA: spinal anaesthesia; SD: standard deviation; CI: confidence interval [18,20,21]

Change in IPSS: The change in IPSS was reported in 3 studies [19-21], enrolling 1032 patients (Figure 8). There was no statistically significant difference in change in IPSS between the GA and SA cohorts (MD - 0.11, 95% CI -1.96 to 1.75, P=0.91). High heterogeneity was detected amongst the included studies (I2=84%, P=0.002).

**Figure 8:**
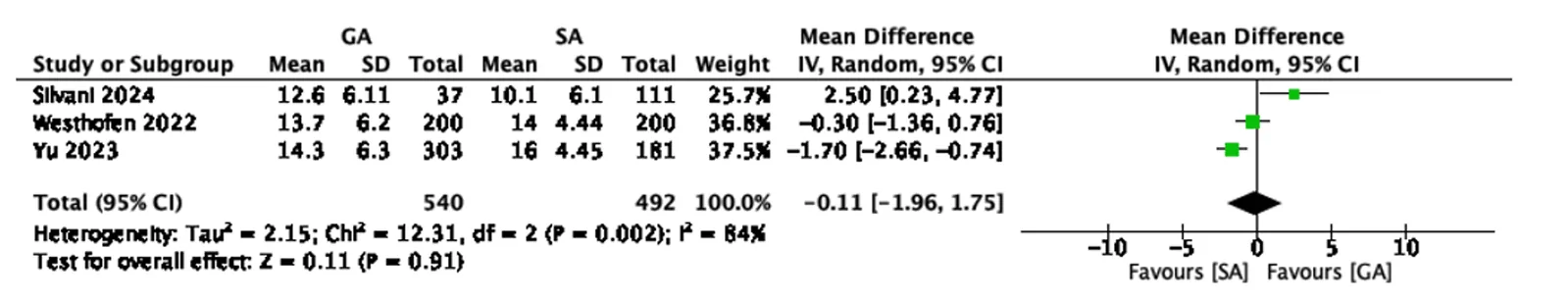
Forest plot of change in IPSS GA: general anaesthesia; SA: spinal anaesthesia; SD: standard deviation; CI: confidence interval; IPSS: International Prostate Symptom Score [19-21]

Change in QMax: The change in QMax was reported in 3 studies [19-21], enrolling 1707 patients (Figure 9). There was no statistically significant difference in change in QMax between the GA and SA cohorts (MD 0.55, 95% CI -1.52 to 2.63, P=0.60). High heterogeneity was detected amongst the included studies (I2=77%, P=0.01).

**Figure 9:**
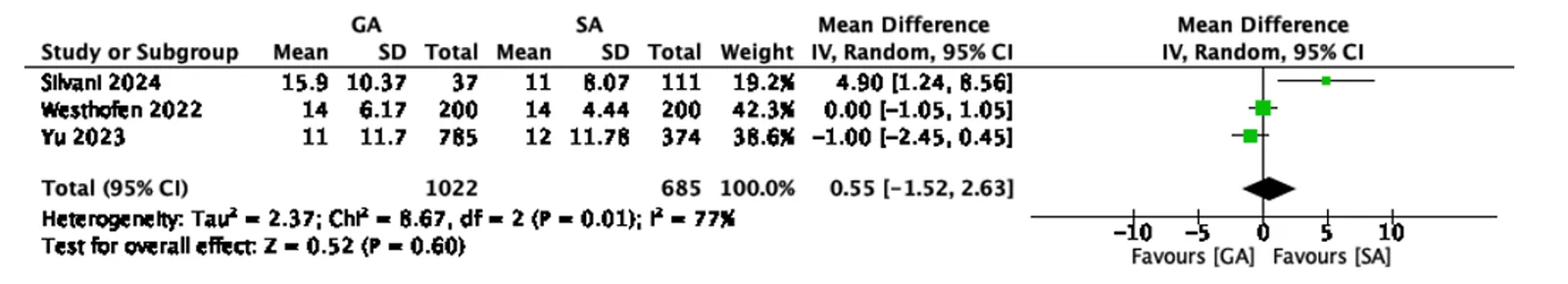
Forest plot of change in QMax GA: general anaesthesia; SA: spinal anaesthesia; SD: standard deviation; CI: confidence interval [19-21]

Change in PVR: The change in PVR was reported in 3 studies [19-21], enrolling 1707 patients (Figure 10). There was no statistically significant difference in change in PVR between the GA and SA cohorts (MD 0.00, 95% CI -8.80 to 8.80, P=1.00). Low heterogeneity was detected amongst the included studies (I2=0%, P=1.00).

**Figure 10:**
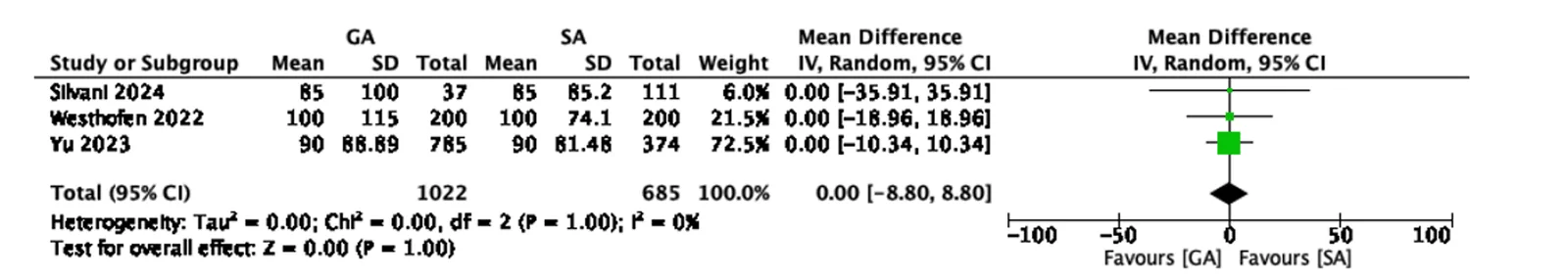
Forest plot of change in PVR GA: general anaesthesia; SA: spinal anaesthesia; SD: standard deviation; CI: confidence interval; PVR: post-void residual volume [19-21]

Sensitivity Analysis

The use of a fixed-effects model, instead of a random-effects model, shifted the Hb drop outcome to statistical significance, favouring SA (higher Hb drop in GA cohort (MD 0.18, 95% CI 0.09 to 0.26, P=0.0001). Leave-one-out sensitivity analysis was able to be performed only for the operative time outcome, due to the outcome being reported by 4 studies [18-21]. Leaving out Haehn et al. [18], or Yu et al. [21], resulted in this outcome changing from statistically significant to not statistically significant (p>0.05).

Discussion

We performed a systematic review of the literature and meta-analysis of reported outcomes to compare the outcomes of patients having a general or SA for HoLEP procedure for lower urinary tract symptoms. We included 4 comparative studies [18-21], enrolling a total of 2323 patients. Though we found non-robust favourability to SA with regard to operative time, our analyses suggest that there were no other statistically significant differences in intra-operative or post-operative outcomes when comparing GA to SA. The between-study heterogeneity was moderate for most reported outcomes, while the quality of available evidence was moderate, though lacking in high-quality randomised controlled trials (RCTs).

To our knowledge, our analysis is the only review comparing GA and SA when performing HoLEP. However, SA is a well-established anaesthetic modality when performing TURP [22], with the benefit of early recognition of TUR syndrome associated with monopolar TURP [23]. Darwish et al. performed a large propensity-matched comparison of GA versus neuraxial anaesthesia (spinal, epidural and combined spinal-epidural), including 28,486 patients [24]. They found neuraxial anaesthesia to be a safe and effective alternative to GA, and indeed found a lower incidence of post-operative reintervention and sepsis rates with neuraxial anaesthesia.

Our findings further support that SA remains a viable alternative to GA for prostate surgery, and show no statistically significant differences for intra-operative outcomes during HoLEP, including resected weight and Hb drop. However, our analysis found a statistically significant difference in operative time, with those who had SA having a shorter procedure (P=0.03). One potential explanation for this may be as a result of an increased proportion of anticoagulated patients having a GA rather than SA. For example, Yu et al. included 16% of patients having a GA on anticoagulation/antiplatelet therapy, compared to 10% in the SA population (P=0.001) [21]. An additional intra-operative haemostasis effort may likely have contributed to the additional operative time.

It should be noted that the HoLEP learning curve is notoriously challenging [25], even for surgeons experienced in performing TURP, with a plateau on learning curve assessment seen in roughly 30-40 cases [26]. A multicentre evaluation of the HoLEP learning curve found that even in a prospective training structure, almost half of the 9 centres elected to discontinue their involvement in the HoLEP training programme due to complications or ongoing issues [27]. One particular consideration relevant to our study is operative time. Naturally, operative time is longer earlier in the HoLEP learning curve. While time limitations in SA can be overcome, in such cases there may be a preference for GA, particularly to suit and relieve the time pressures of a surgeon relatively new to HoLEP, or when prolonged enucleation time is anticipated.

Our review and reported outcomes have limitations. Firstly, while our search found 4 studies which fit our inclusion criteria, many of our analysed reported outcomes are based on the published results of just 3 studies. The studies included were retrospective analyses or propensity-matched studies. Ideally, further high-power studies, such as RCT’s, with comparable demographics including anticoagulated status, would be performed in the future.

Our results show SA represents a safe and viable option for anaesthesia when performing HoLEP. However, appropriate case selection remains important. SA cases could therefore be considered for surgeons experienced in performing HoLEP, and in those for whom an extended enucleation time is not anticipated.

## Conclusions

Spinal anaesthesia appears to be a safe alternative to general anaesthesia, with no significant differences in intra- or post-operative outcomes. The utilisation of spinal anaesthesia could allow for the utilisation of HoLEP in a cohort of patients considered too high-risk for general anaesthesia. However, surgeon experience, prostate size and the anticipated enucleation time should be factored in when making such decisions. Further studies are required, particularly comparing experienced to inexperienced HoLEP surgeons, and with a variety of prostate sizes.

## Appendices

**Table 3:** Literature search strategy * This search strategy was adopted for the following databases: MEDLINE, PubMed, EMBASE, and Google Scholar

Search no.	Search strategy^*^
#1	MeSH descriptor: [holmium laser enucleation of prostate] explode all trees
#2	Holmium laser enucleation of prostate : TI,AB,KW
#3	MeSH descriptor: [HoLEP] explode all trees
#4	HoLEP: TI,AB,KW
#5	#1 OR #2 OR #3 OR #4
#6	MeSH descriptor: [general anaesthesia] explode all trees
#7	General anaesthesia : TI,AB,KW
#8	MeSH descriptor: [GA] explode all trees
#9	GA: TI,AB,KW
#10	MeSH descriptor: [spinal anaesthesia] explode all trees
#11	Spinal anaesthesia: TI,AB,KW
#12	MeSH descriptor: [SA] explode all trees
#13	SA: TI,AB,KW
#14	#6 OR #7 OR #8 OR #9 OR #10 OR #11 OR #12 OR #13
#15	MeSH descriptor: [benign prostatic hyperplasia] explode all trees
#16	benign prostatic hyperplasia: TI,AB,KW
#17	MeSH descriptor: [bladder outlet obstruction] explode all trees
#18	bladder outlet obstruction: TI,AB,KW
#19	#15 OR #16 OR #17 OR #18
#20	#5 AND #14 AND #19
